# Alginate Biosynthesis of *Pseudomonas aeruginosa*: Comparison of Coating and Film Formation Properties of Bacterial Alginate With Brown Algae Alginate in Beef Thigh Meat Shelf Life

**DOI:** 10.1002/fsn3.71804

**Published:** 2026-04-20

**Authors:** Leila Soleiman Garmabaki, Behnaz Bazargani‐Gilani, Ali Goudarztalejerdi

**Affiliations:** ^1^ Department of Food Hygiene and Quality Control, Faculty of Veterinary Medicine Bu‐Ali Sina University Hamedan Iran; ^2^ Department of Pathobiology, Faculty of Veterinary Medicine Bu‐Ali Sina University Hamedan Iran

**Keywords:** algal alginate, bacterial alginate, beef thigh meat, edible coating, shelf life

## Abstract

This research aimed to compare the coating and film‐forming properties of the bacterial alginate (BA) produced by over‐producer alginate bacteria ( *Pseudomonas aeruginosa* ) and brown algae alginate (AA) in the shelf life enhancement of beef thigh meat during refrigerated storage. Fourier Transform Infrared Spectroscopy (FT‐IR) spectra of bacterial alginate (BA) and AA were similar in appearance and confirmed the identity of alginate. According to Scanning Electron Microscopy (SEM) images, BA particles exhibited a rough surface, irregular polygonal structures with sharp sides and corners in comparison with AA. In contrast, AA had a smooth, more uniform surface and filamentous structure compared with BA. The AA film, with a thickness of 0.20 ± 0.04 mm, tensile strength of 41.87 ± 1.2 MPa, elongation at break of 60.53%, and Young's modulus of 102.98 MPa, exhibited significantly superior mechanical properties (*p* ≤ 0.05) compared with the BA film. Also, BA‐based edible coatings significantly (*p* ≤ 0.05) decreased the microbial populations (total viable count, psychrotrophic bacteria, *Enterobacteriaceae*, and yeast/mold), pH, and total volatile basic‐nitrogen (TVB‐N) changes of coated beef slices compared with the AA treatments during 9 days of refrigerated storage. Furthermore, sensory analyses revealed that BA groups were the most superior treatments in the stability of sensory characteristics (color, odor, and overall acceptability) of the samples. The current study results indicated that the BA coatings could significantly (*p* ≤ 0.05) elongate the shelf life of refrigerated beef thigh meat up to 9 days compared to AA. These findings highlight the higher potential of BA‐based edible coating as natural and effective packaging than AA in same concentration. Therefore, considering the mechanical characteristics of AA and preserving properties of BA, it is suggested to use a mixture of these two alginates in an appropriate ratio for ideal food packaging and formulation.

## Graphical abstract

Current study results indicated that *Pseudomonas aeruginosa* alginate and brown algae alginate exhibited significant differences (*p* ≤ 0.05) in the morphology and physicochemical characteristics. The mechanical features of brown algae alginate films were significantly (*p* ≤ 0.05) more superior to *Pseudomonas aeruginosa* alginate films. According to the obtained findings, the *Pseudomonas aeruginosa* alginate coatings could significantly (*p* ≤ 0.05) elongate the shelf life of refrigerated beef thigh meat up to 9 days compared to brown algae alginate.

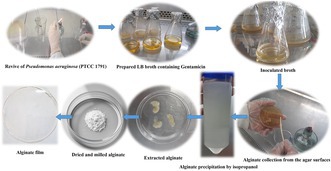

## 1 Introduction

Beef thigh meat, including the main muscles such as the semitendinosus, *Biceps femoris*, and semimembranosus, is one of the most important parts of beef meat in the food industry due to its high nutritional value, tender texture, and widespread use in fresh products, such as steaks and kebabs as well as processed products, such as sausages and burgers. Global demand for beef meat has reached 72 million tons in 2023, with a significant share of thigh meat due to its quality and multipurpose use (Yang et al. 2025). Beef thigh meat is favored by consumers due to its high nutritional value and desirable sensory characteristics, including color and palatable taste. This product is a rich source of micronutrients, such as vitamin B12, heme iron, zinc, selenium, and essential fatty acids, which play a key role in overall health, immune system function, and anemia prevention (Cabrera and Saadoun 2014). However, high moisture and nutrient content have led to its susceptibility to microbial spoilage and lipid oxidation. Recently, the use of biodegradable packaging in food products has been considered (Ghanbar Soleiman Abadi et al. 2024). The complete biodegradability of alginate biopolymer offers a sustainable alternative to conventional plastic packaging with low oxygen permeability, playing a significant role in reducing environmental waste. The building blocks of alginate are β‐D‐Mannuronic acid (M) and α‐L‐Guluronic acid (G) units. This linear anionic polysaccharide is widely used in the packaging of meat due to its biocompatibility, biodegradability, and ability to form resistant films. This biopolymer, with its barrier properties against oxygen and moisture, inhibits oxidation and microbial growth and finally preserves the sensory properties of meat (Cazón et al. 2017; Mohamed et al. 2020; Senturk Parreidt et al. 2018). Alginate creates a three‐dimensional network of ionic bonds between Guluronic (G) blocks and divalent cations (Ca^2+^) based on the “egg box” model. Alginate is produced by brown algae with scientific names of *Macrocystis pyrifera* and *Laminaria hyperborea* and bacteria, such as *Pseudomonas aeruginosa* , as well (Rhein‐Knudsen et al. 2015). Algal alginate use in the food industry is dominant due to their low production cost (0.5–1 $/kg) and wide availability; but, the lack of antimicrobial activity and impurities, such as polyphenols and sulfates (1%–2%) have decreased bioactivities in the packaging (McHugh 2003). Bacterial alginate is produced in controlled bioreactors with pH of 7.0, temperature of 30°C–37°C, dissolved oxygen of 20%–30% with higher purity (99%), and tunable properties; however, its production cost (5–10 $/kg) is higher than algal alginate. Acetylation degree (10%–20%) of bacterial alginate leads to considerable antimicrobial activities, higher viscosity, pseudo‐plastic behavior, flexibility, and decreases gel strength up to 20%. High purity and condensed structure of bacterial alginate improve its efficiency in industrial applications (Moradali et al. 2017). Since there is no comprehensive study about the comparison of coating and film formation properties of bacterial alginate with algal alginate in a food model, this study was aimed at comparing the efficiencies of alginate produced by *Pseudomonas aeruginosa* with brown algae alginate coatings and films in the shelf‐life improvement of beef thigh meat during refrigeration conditions.

## 2 Materials and Methods

### 2.1 Materials

*Pseudomonas aeruginosa* PTCC 1791 was provided by the Iranian Research Organization for Science and Technology (IROST, Tehran, Iran). Brown algae alginate was purchased from the Sigma Company (Darmstadt, Germany). Calcium chloride, isopropanol, boric acid, selenium dioxide, sodium hydroxide, sulfuric acid, glycerol, methanol, ethanol, nutrient agar, nutrient broth, Luria‐Bertani (LB) broth, *Pseudomonas* isolation agar (PIA), *Pseudomonas* isolation broth (PIB), cetrimide agar, peptone water, violet red bile glucose agar, plate count agar, and rose bengal chloramphenicol selective agar were provided by Merck Company (Darmstadt, Germany).

### 2.2 Revive of *Pseudomonas aeruginosa*

*Pseudomonas aeruginosa* PTCC 1791 was inoculated in 100 mL of Nutrient broth and incubated at 37°C for 24 h, aerobically. A 25% glycerol stock of *P. aeruginosa* PTCC 1791 was prepared and stored at −80°C for future investigations (Valentine et al. 2020).

### 2.3 Cultivation of *Pseudomonas aeruginosa*

Bacteria were cultured by surface and double‐layer pour‐plate method for accurate count of surface and subsurface colonies, respectively (ISO 2013). In the surface method, 100 μL of bacterial suspension (10^6^ CFU/mL) was spread on Nutrient agar. In the pour plate method, 1 mL of the suspension was mixed with 15 mL of molten agar (45°C). After drying the first layer, 10 mL of molten agar was added as a second layer. The plates were incubated at 37°C for 24–48 h.

### 2.4 Bacterial Alginate Preparation

**FIGURE 1:**
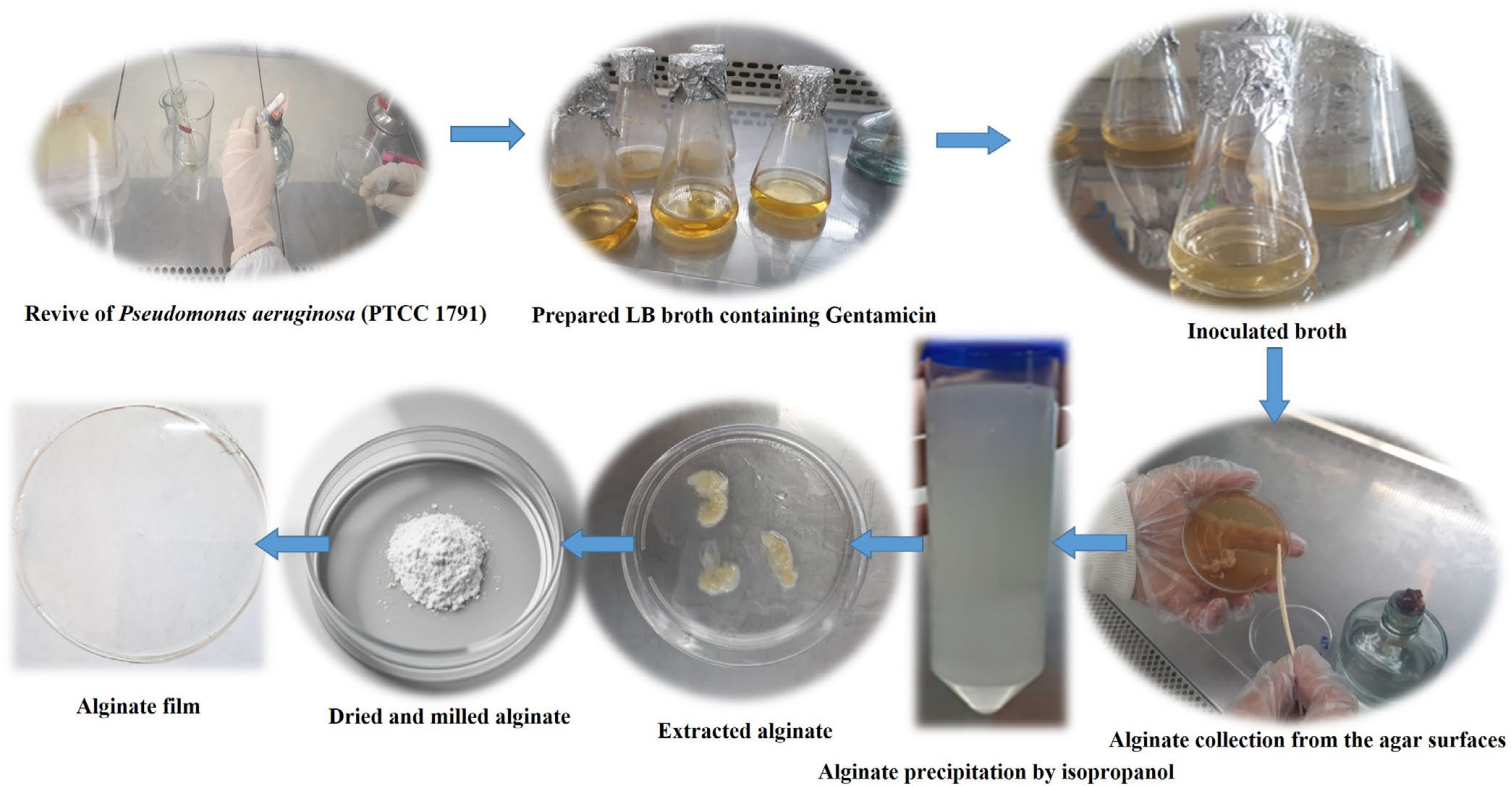
Alginate extraction steps from *Pseudomonas aeruginosa*.

Alginate produced by *P. aeruginosa* PTCC 1791 grown on solid medium was harvested according to previously described methods (Remminghorst and Rehm 2006; Wang et al. 2016), with modifications. A loop full of frozen culture stock was inoculated in PIB and incubated at 37°C for 24 h. Then, 1 mL of this culture was added to LB broth containing Gentamicin (300 μg/mL) and incubated at 37°C for 24 h. Next, cells of an overnight culture were washed twice with saline and suspended to an optical density (OD600 nm) of 30. The 100 μL of suspended bacteria were plated onto PIA medium containing Gentamicin (1% *W*/*V*) and incubated at 37°C for 72 h. Then, the biomass was scrapped from agar surfaces and dissolved in 150 mL of sterile saline and shaken at 150 rpm for 60 min. Cells were removed by centrifugation (8000× *g*, 10 min, 4°C) in a refrigerated centrifuge and the alginate in the supernatant was precipitated with 1 volume ice‐cold isopropanol. After drying the extracted alginate under a laminar flow hood (LCB‐H1300, Scitek Company, country) in sterile conditions, it was milled and stored in sterile containers at 4°C for next analyses (Remminghorst and Rehm 2006; Wang et al. 2016) (Figure 1).

### 2.5 Alginate Analysis

Alginate harvested from *P. aeruginosa* PTCC 1791 (BA) and algal alginate (AA), properties and composition were assessed by Fourier Transform Infrared Spectroscopy (FT‐IR) and Scanning Electron Microscope (SEM) images of the alginates.

#### 2.5.1 Fourier Transform Infrared Spectroscopy (FT‐IR)

In order to prepare the samples for FT‐IR analysis, some of the alginates were mixed with KBr. Then, they were pounded in a porcelain mortar. After preparing a tablet of the samples with a thickness of 1 mm using a tablet making machine, FT‐IR analysis was carried out by a Fourier Transform Infrared Spectrometer (Helious, England) at the range of 500 to 4000 cm^−1^ with the resolution of 4 cm^−1^ (Davarcioğlu 2010).

#### 2.5.2 Scanning Electron Microscope (SEM) Images of Alginates

SEM images of the alginates were prepared by an electron microscope device (JSM‐840A, Japan) at the magnifications of 100×, 300×, and 1000×. The alginate samples were installed on a bronze stand and coated with gold. An accelerating voltage of 10 kV was used to observe the samples (Aly et al. 2024; Jebel et al. 2025).

### 2.6 Preparation of Bacterial and Algal Alginate‐Based Films

Bacterial and algal alginate powders (2.5% *W*/*V*) were dissolved in distilled water. Glycerol was used (0.3 g/g alginate) as a plasticizer. Calcium chloride (0.05 g/g alginate) was added to the mixture for crosslinking reactions. The obtained solution was stirred under 70°C for 20 min. After homogenizing the solutions by homogenizer at 12,200 rpm for 3 min, the solutions were sonicated for degassing for 8 min. The casting method was used for the film formation; so, the film solutions were poured on glass plates in an incubator chamber at 25°C with 50% relative humidity (RH) for 72 h. After drying, the formed films were gently peeled off. All prepared films were conditioned besides saturated magnesium nitrate for 48 h (RH of 50% at 25°C) (Kim et al. 2018).

### 2.7 Film Analyses

#### 2.7.1 Mechanical Analysis

Tensile strength (TS), elongation at break (EB), and Young's modulus (YM) were measured by a texture analyzer device (Sanam/STM‐5). Strips of the films were cut to a width of 25 mm and a length of 100 mm. The distance between the two jaws of the device was set to 5 cm and the loading rate was set to 20 mm/min (ASTM 2012).

#### 2.7.2 Thickness Measurement

A digital micrometer (IP65 Alpa Exacto, Milan, Italy) with an accuracy of 0.001 mm was used for the thickness measurement of the films (ASTM 2012).

### 2.8 Preparation of Alginate‐Based Coating Solutions

Bacterial alginate, algal alginate, and chloride calcium powder were mixed to distilled water (2% *W*/*V*) and stirred at 50°C during 30 min for complete dissolution separately (Tavassoli‐Kafrani et al. 2016).

### 2.9 Preparation of Treatments

Fresh beef thigh meat was provided from the meat processing plant, Hamedan, Iran. The samples were transported to the laboratory on ice flakes. Under complete sterile conditions, the beef thigh meat was cut into 10‐g pieces (2 × 4 × 4 cm) in aseptic condition and immersed in coating solutions for 1 min. After forming the first layer, they were immersed in chloride calcium solution (2%) during 30 s for crosslink reactions. Then, the samples were placed in sterile zip packs and stored in the refrigerator (4°C ± 1°C), and microbiological, chemical, and sensory analyses were performed on 0‐, 3‐, 6‐, and 9‐day intervals. Coated treatments were as follows: uncoated meat (control), coated meat by algal alginate coating (AA), and coated meat by bacterial alginate coating (BA).

### 2.10 Microbial Analysis of Treatments

For the microbial analysis, 10 g of the samples was mixed with 90 mL of peptone water (0.1%) and homogenized by a stomacher (Bag Mixer 400, Interscience, France) at 250 rpm for 60 s. After preparing 10‐fold serial dilutions, the samples were cultured on related mediums. So, Plate Count Agar was used for total viable count (TVC) of mesophilic and psychrotrophic bacteria. Then, Violet Red Bile Glucose agar and Rose Bengal Chloramphenicol Selective agar were applied for *Enterobacteriaceae* and yeast‐mold enumeration, respectively. The microbial findings were reported as Log_10_ CFU/g meat (Yousef et al. 2022).

### 2.11 Chemical Analysis of Treatments

#### 2.11.1 pH

After mixing 5 g of the samples with 25 mL of the distilled water, the obtained solutions were homogenized for 60 s. Then, a pH meter (E520, Metrohm Herisau, Bern, Switzerland) was used for reading pH values (Ghanbar Soleiman Abadi et al. 2024).

#### 2.11.2 TVB‐N

The total volatile basic‐nitrogen (TVB‐N) values of the treatments were measured by the method of Fernández et al. (2009). So, 10 g of the samples, 300 mL of distilled water, and 2 g of MgO were distilled in a Kjeldal flask. The condensed vapors reacted with boric acid (2%) containing reagent in the Erlenmeyer flask. After the appearance of a green color, the sample was titrated with sulfuric acid (0.1 N). Then, TVB‐N value was calculated by following the formula:

$$\mathrm{TVB}-N\;\left(\mathrm{mg}/100g\right)=\text{Volum of consumed acid}\times 14$$

### 2.12 Sensory Analysis

A group consisting of 20 (10 females and 10 males) between 25 and 35 years old from Food Hygiene and Quality Control Department members, laboratory‐trained and experienced judges in meat assessment, conducted organoleptic analysis. All of these judges who assessed the sensory properties of samples had participated in training meetings to learn about the sensory features of meat. Treated meats were analyzed in white porcelain trays by panelists. The panelists were not informed about the experimental approach, and the samples were blind‐coded with 3‐digit random numbers. Fresh meat was considered as the reference. The sensory characteristics (odor, color, and overall acceptability) of the treatments were evaluated. The panelists analyzed samples on a 5‐hedonic scale, including 1: very unsatisfactory, 2: unsatisfactory, 3: acceptable, 4: satisfactory, and 5: very satisfactory (Ghanbar Soleiman Abadi et al. 2024).

### 2.13 Statistical Analysis

All experiments were carried out in triplicates. SPSS (IBM SPSS statistics 21) and Excel software were considered for data analysis and graph designing. Variance analysis (ANOVA) and the Tukey test at the significance set of *p* ≤ 0.05 were considered to show the differences among the treatments. The analyzed data were exhibited as mean values ± standard deviations (SD).

## 3 Results and Discussion

### 3.1 Ft‐IR

**FIGURE 2:**
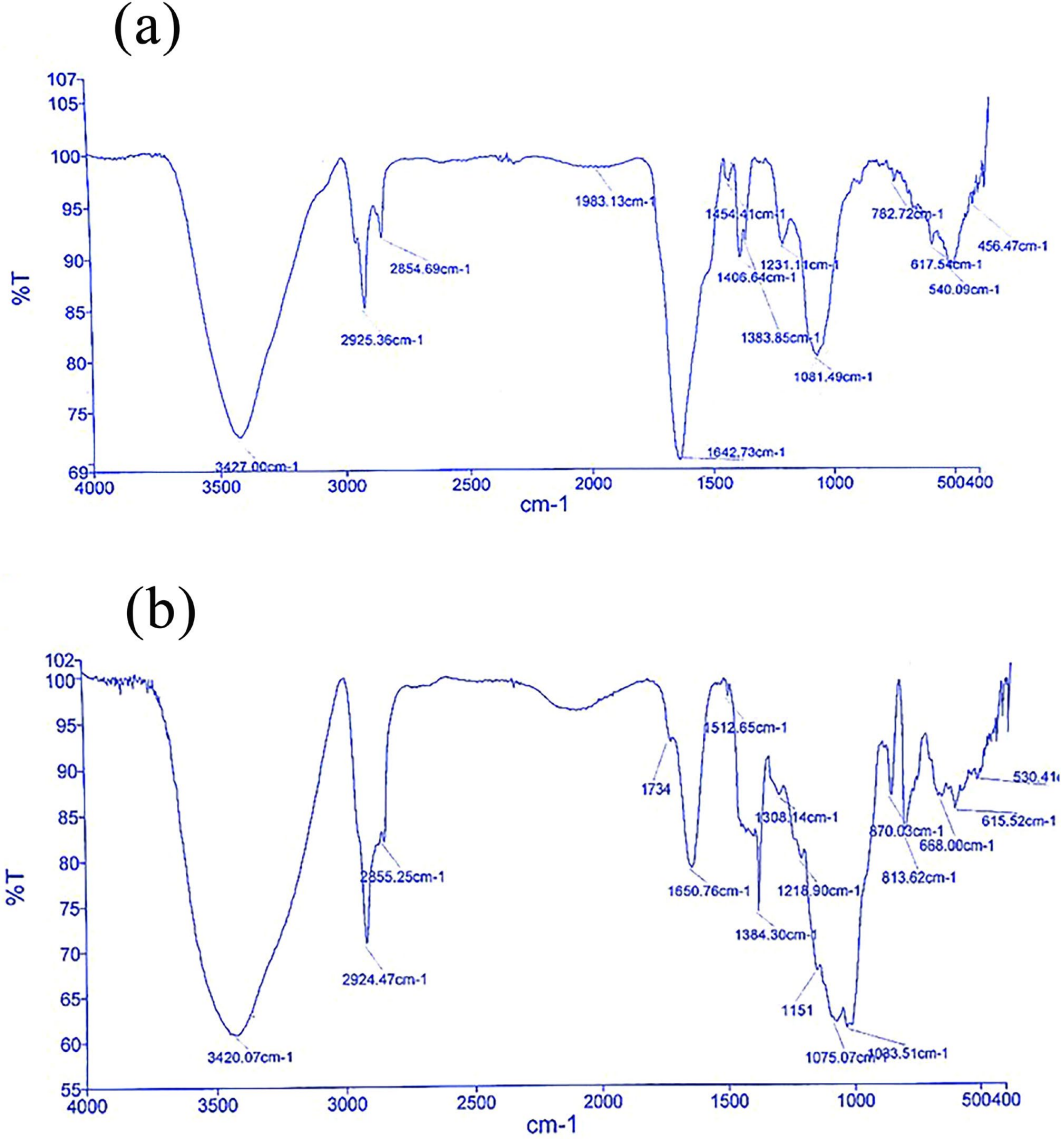
(a, b) FT‐IR spectrum of BA (a) and AA (b). Treatments: BA (bacterial alginate), AA (algal alginate).

FT‐IR spectroscopy was used for the analysis of bacterial and algal alginate chemical groups and structures. Figure 2a,b illustrates FT‐IR spectra of BA (Figure 2a) and AA (Figure 2b). In general, both spectra are similar in appearance and indicate that these samples are alginate. The broad peaks at 3427 in BA and 3420.07 cm^−1^ in AA spectra are related to OH‐stretching vibrations (Fenoradosoa et al. 2010). The CH aliphatic bonding was detected at the range of 2925.36 cm^−1^ and 2924.47 cm^−1^ in BA and AA spectra, respectively. The acetyl group is defined as a functional group characterized by the presence of a carbonyl (C=O) and a methyl (CH_3_) group. The peaks at 1642.73 cm^−1^ in BA and 1650.76 cm^−1^ in AA spectra represent C=O stretching vibration, which is remarkably sharper for BA. This peak can be correlated to the C=O of acetyl group (−C(=O) − CH_3_). The sharper peak of BA can obviously explain the higher antimicrobial activity of bacterial alginate compared with the AA (Fertah et al. 2017). The C‐O‐stretching vibrations were observed at 1000–1200 cm^−1^ in both spectra, which were weaker for BA (Ardalan et al. 2018). Also, these spectra showed that the peak values at primary wavenumbers for AA are close to BA. Therefore, according to the FT‐IR spectra of both alginates, the successful harvesting of BA was confirmed.

### 3.2 Scanning Electron Microscope (SEM) Images of Alginates

**FIGURE 3:**
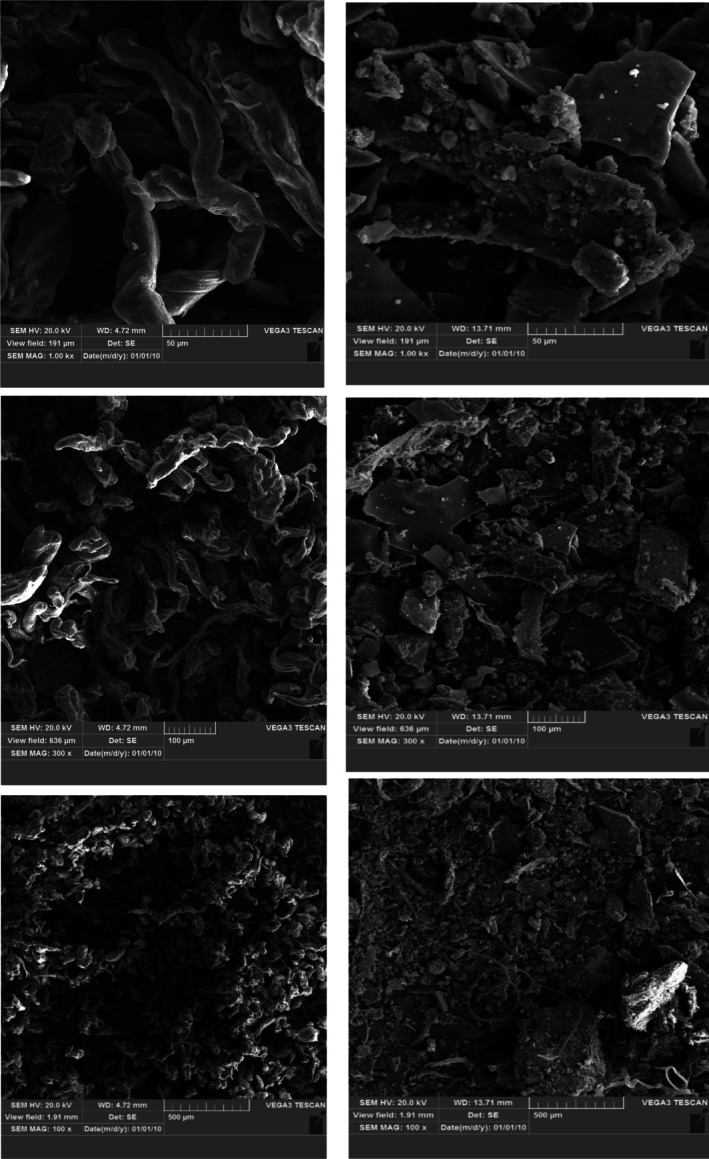
SEM images of AA (left column) and BA (right column) at the magnifications of 1000×, 300×, and 100×.

Figure 3 Illustrates SEM images of AA (left column) and BA (right column) at the magnifications of 100×, 300×, and 1000×. According to the obtained images, BA exhibited a rough surface, irregular polygonal structures with sharp sides and corners in comparison with AA (Figure 3). In contrast, AA had a smooth, more uniform surface and filamentous structure in the SEM image compared with BA. In consistency with our findings, Sayin et al. (2022) observed a round filamentous and flat pattern in SEM images of algal alginate ( *Sargassum vulgare* ). While Olad and Farshi Azhar (2014) reported that the SEM micrograph for alginate revealed irregular spherical granules in the size range of 5–45 μm, the surface was quite smooth. In other studies, sharp‐edged, rough, and irregular particles of alginate were observed in SEM images (Daemi and Barikani 2012; Shabbir et al. 2017). Aly et al. (2024) reported that the surface of sodium alginate is rough and it appears rock‐like in SEM images. Also, they observed that sodium alginate pore size decreased by the γ‐irradiation; so, the macromolecular structure disappeared and the surface became smoother.

### 3.3 Film Analyses

#### 3.3.1 Mechanical Analysis

Mechanical properties of the AA and BA films were presented in Table 1. According to the obtained findings, tensile strength (41.789 MPa), elongation at break (60.53%) and Young's modulus (102.98 MPa) of AA films were significantly (*p* ≤ 0.05) higher than BA films (35.24 MPa, 45.32%, and 85.67 MPa). In other words, AA films showed stronger mechanical characteristics compared with BA films that can be attributed to the more condensed molecular structure and higher hydrogen bonds in AA. Therefore, AA films are more ideal candidates against mechanical stresses in food packaging during the storage period.

**TABLE 1:** Mechanical properties of the alginate films. Treatments: BA (bacterial alginate film), AA (algal alginate film). Different letters indicate significant differences between treatments (*p* ≤ 0.05).

Films	Tensile strength (MPa)	Young's modulus (MPa)	Elongation at break (%)	Thickness (mm)
BA	35.24 ± 0.02^b^	85.67 ± 0.05^b^	45.32 ± 0.01^b^	0.15 ± 0.05^b^
AA	41.78 ± 0.06^a^	102.98 ± 0.07^a^	60.53 ± 0.08^a^	0.20 ± 0.04^a^

The key difference between algal and bacterial alginates lies in the ratio of D‐mannuronate to L‐guluronate (M/G ratio) and the distribution of G and M blocks in the polymer chain. Algal alginates typically have a higher G/M ratio (> 1), indicating a predominance of Glucuronic acid (G) units. This higher ratio results in the formation of longer GG blocks, which increases the stiffness and strength of the gel. In contrast, bacterial alginate has a lower G/M ratio (< 1) and predominant Mannuronic acid (M) units, which creates more flexible films in comparison to AA. (Valentine et al. 2020) showed that the produced alginate by an attenuated strain (MucE‐5PGN) of *Pseudomonas aeruginosa* was rich in Mannuronic acid, which was confirmed by HPLC analysis and led to the higher flexibility and adhesion of bacterial alginate compared with the algal alginate. Whitney et al. (2011) suggested that these differences in G/M ratios are due to different biosynthetic pathways in bacteria, such as *Pseudomonas aeruginosa* and brown algae; so, biosynthetic enzymes, such as the outer membrane protein (AlgE) in bacteria preferentially produce M blocks. Valentine et al. (2020) reported that *Pseudomonas aeruginosa* (MucE‐5PGN strain) produced alginate with high purity and uniform molecular weight by deleting virulence‐related genes (such as exotoxin A) and overexpressing the *MucE* gene which is more ideal for commercial applications. Algal alginate is produced naturally in the cell wall of algae and is extracted by chemical methods.

#### 3.3.2 Thickness

Thickness of the studied films was exhibited in Table 1. AA films showed significantly (*p* ≤ 0.05) higher thickness (0.2 ± 0.04 mm) compared with the BA films (0.15 ± 0.01 mm). Previous studies reported that this difference is attributed to the higher viscosity of AA solution (1000–2000 mPa.S) compared with the BA solution (400–600 mPa.S), which can be correlated to the higher ratio of Glucuronic acid to Mannuronic acid (G/M > 1) in the AA structure. Higher thickness of AA film creates a stronger barrier against oxygen and moisture penetration in food packaging, which leads to lower lipid oxidation and microbial growth of the food during the storage period (Valentine et al. 2020; Zhang et al. 2021).

### 3.4 Microbial Analysis

#### 3.4.1 Total Viable Count

**FIGURE 4:**
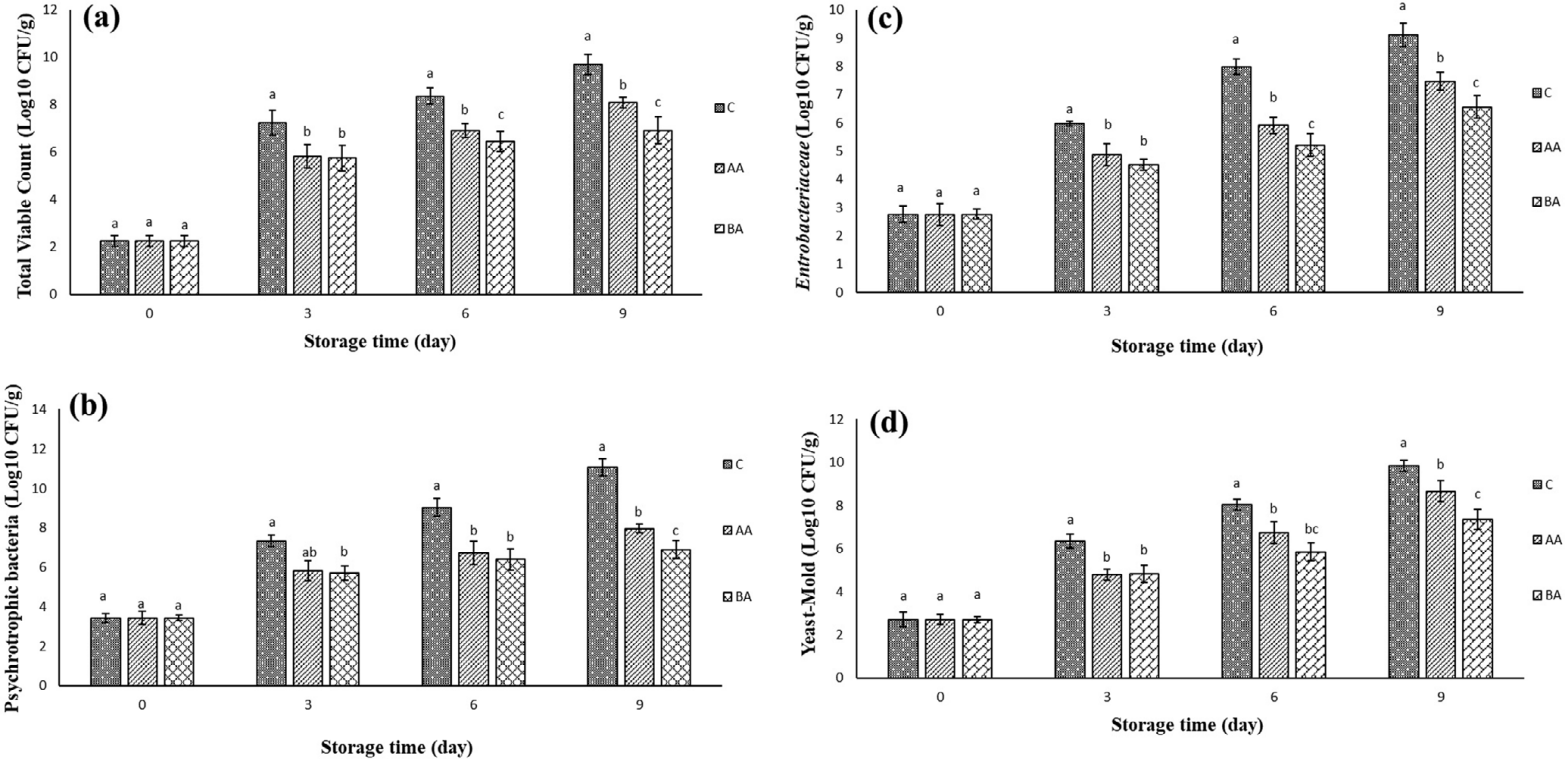
(a–d) Changes of microbial populations (total viable count [a], psychrotrophic bacteria [b], *Enterobacteriaceae* [c], and yeast‐molds [d]) of the treated samples. Treatments: C (control, un‐coated beef slices), BA (coated beef slices by bacterial alginate), and AA (coated beef slices by algal alginate). Different letters within the same day (a, b, c, etc.) indicate a statistically significant difference (*p* ≤ 0.05).

Changes in total viable count (TVC) of the coated beef thigh slices are illustrated in Figure 4a. The initial TVC of the studied samples was 2.25 Log CFU/g (Figure 4a) on day 0. By increasing the storage period, the TVC of all treatments increased; so, the control group went beyond the standard level (7 Log CFU/g) on the 3rd day of the storage time (ICMSF 1986). While the AA coated slices remained in the consumable range (< 7 Log CFU/g) on the 6th day of the storage period. But, BA treatment (6.92 Log CFU/g) never exceeded the allowable limit of TVC until the end of the storage time. BA and AA coatings significantly (*p* ≤ 0.05) decreased microbial growth compared with the control. BA (6.92 Log CFU/g) treatment significantly (*p* ≤ 0.05) showed the lowest TVC compared with other treatments, followed by AA (8.07 Log CFU/g) and control (9.69 Log CFU/g) groups, respectively at the 9th day of the storage time (Figure 4a). The BA treatment demonstrated superior antimicrobial activities among the others during the storage period.

#### 3.4.2 Psychrotrophic Bacteria

One of the main microflora of meat is gram‐negative psychrotrophic bacteria (PB) that quickly spoil aerobically‐packaged fresh meat under refrigeration conditions (Ghanbar Soleiman Abadi et al. 2024). Figure 4b represents the changes in the PB population of the studied samples during storage time. There were no significant differences in PB population among the treatments on day 0. With increasing storage duration, all treatments exhibited an increasing trend. So, control samples went beyond the standard level (7 Log CFU/g) on the 3rd day of storage. Notably, the rate of increase was slower in the coated samples compared with the uncoated ones. BA treatment showed the lowest psychrotrophic bacteria (6.89 Log CFU/g) count among the other groups that followed AA (7.95 Log CFU/g) group throughout the storage period (Figure 4b). It is worth mentioning that BA treatment never exceeded the standard limit (> 7 Log CFU/g) of PB during 9 days of the storage period.

#### 3.4.3 Enterobacteriaceae

*Enterobacteriaceae*, facultative anaerobic bacteria, are considered microbial flora in the types of meat that spoil them at chilled temperatures (ICMSF 1986). *Enterobacteriaceae* population of the treated slices was exhibited in Figure 4c. No statistically significant differences in *Enterobacteriaceae* population were detected among the treatments (*p* > 0.05) on day 0. An increasing pattern was found in all groups over the storage period. Uncoated samples (control) demonstrated the most rapid microbial proliferation. Both alginate coatings (AA and BA) significantly (*p* ≤ 0.05) reduced bacterial growth rates. BA treatment showed superior antimicrobial activities and showed the lowest *Enterobacteriaceae* counts throughout the 9‐day storage period. AA treatment also exhibited significant microbial inhibition compared with the control (Figure 4c). According to the Figure 4c, BA and the uncoated groups significantly (*p* ≤ 0.05) showed the lowest (6.56 Log CFU/g) and highest (9.10 Log CFU/g) *Enterobacteriaceae* population among the others at the end of the storage time, respectively.

#### 3.4.4 Yeast and Mold

Yeast and mold are considered as the microflora of the cold stored food and can easily grow in any conditions (ICMSF 1986). According to Figure 4d, an increasing pattern was found in the yeast and mold enumeration of the treatments during the storage period. However, this increase was significantly (*p* ≤ 0.05) lower in all coated samples (BA and AA) compared with the control sample. The lowest yeast and mold count was observed in the BA treatment throughout the storage period, while the highest yeast and mold population was found in the control sample (Figure 4d). Uncoated samples significantly (*p* ≤ 0.05) showed the highest enumeration (9.84 Log CFU/g) compared with the others, and AA (8.66 Log CFU/g) and BA (7.35 Log CFU/g) were in the next ranks, respectively at the end of the storage period (Figure 4d).

Ghalehjooghi et al. (2023) reported that active packaging nanofiber mats based on gelatin‐sodium alginate could significantly (*p* ≤ 0.05) lower the TVC of wrapped silver carp fillet compared with the control samples during 14 days of the storage time.

One of the distinctive features of bacterial alginate is the presence of acetyl groups in Mannuronic acid units, which are absent in algal alginate. Biosynthesis and secretion of these acetyl groups in BA are regulated by proteins, such as AlgE. They improve antimicrobial activities and adhesion properties of bacterial alginate (Remminghorst and Rehm 2006; Whitney et al. 2011).

Valentine et al. (2020) confirmed that acetylation of Mannuronic acid units in produced alginate by PGN5 increased antibacterial activities against *Escherichia coli* and *Salmonella typhimurium* by increasing hydrogen interactions and reducing oxygen permeability. Therefore, bacterial alginate can create a strong surface contact to food and inhibit oxygen permeability followed by microbial growth in the coated food during storage time (Tong et al. 2023). In contrast, the lack of acetyl groups in algal alginate leads to reduced adhesion and lower flexibility of the alginate coating on the food surface.

Zhang et al. (2021) showed that the agar/sodium alginate (AS) and AS containing ginger essential oil (AS + GEO) coatings elongated the shelf life of chilled beef samples by 6 and 9 days, respectively. They concluded that the designated coatings can be considered active packaging in the shelf life enhancement of fresh beef during refrigerated storage. Smeti et al. (2025) observed that alginate coated beef meat had a higher red and chroma indices compared with the control and irradiated samples. Also, they found that alginate coating significantly (*p* ≤ 0.05) lowered the firmness of beef meat compared with other treatments. They concluded that alginate edible coating is a powerful alternative to irradiation treatment in the shelf life enhancement of beef meat during the storage period. Wu et al. (2024) reported that the ethyl cellulose/gelatin‐based composite nano‐film could significantly (*p* ≤ 0.05) decrease the TVC (5.98 Log CFU/g) of the packed pork to 6 days compared with the control samples during the refrigerated storage period. Another study revealed a significant decrease (*p* ≤ 0.05) of TVC in the gelatin‐wrapped mackerel fillet in comparison with the unwrapped samples during 12 days of the storage period (Sayadi et al. 2025). The superiority of the BA group may be related to its more appropriate adhesion features, lower water vapor permeability, transparency value, and higher antimicrobial activity than the other groups. These properties create an ideal and stable barrier against gases, oxygen, water vapor, and light in the coated and wrapped samples over the storage period.

### 3.5 Chemical Analysis

#### 3.5.1 pH

**FIGURE 5:**
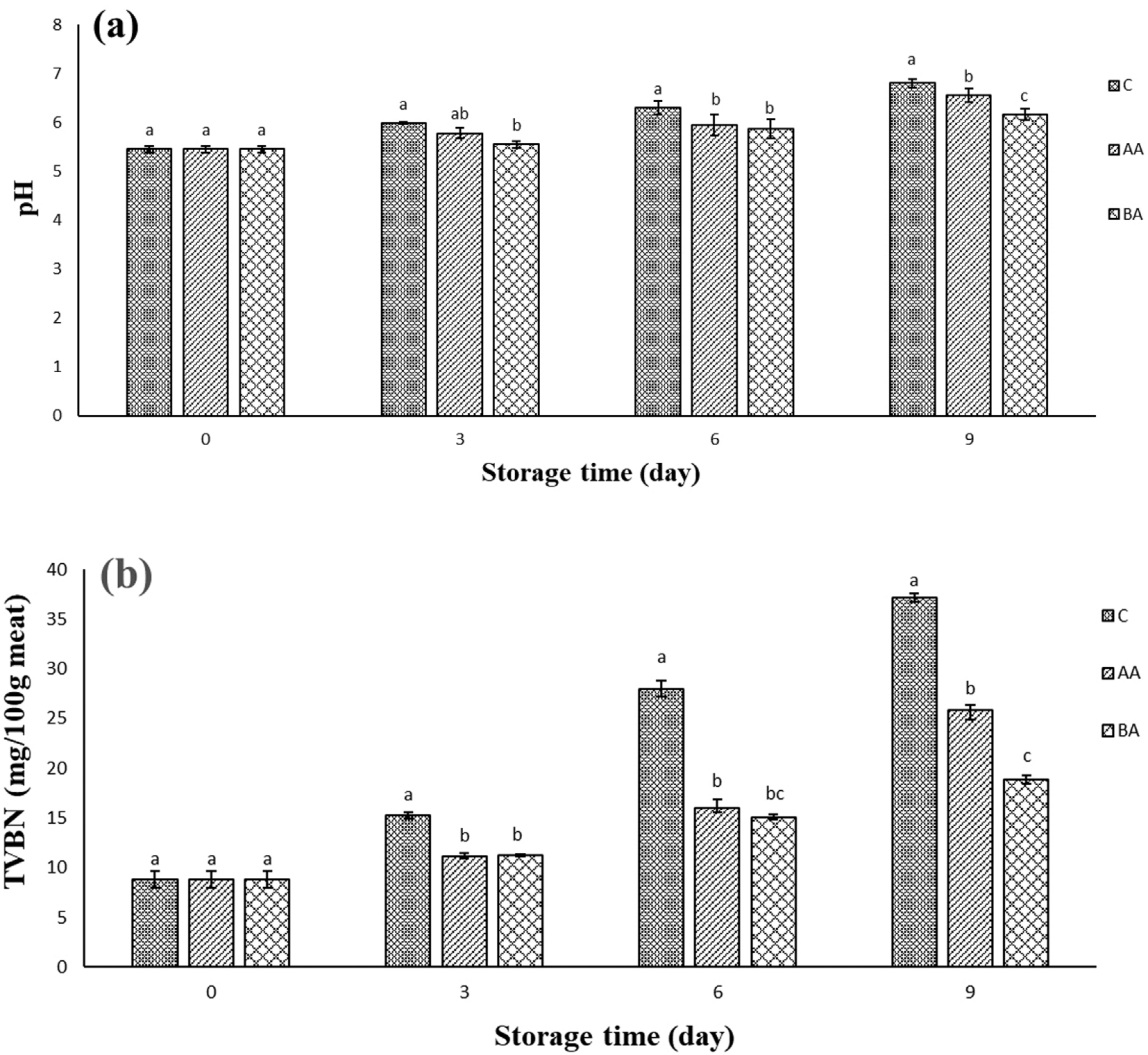
Changes of pH (a) and TVB‐N (b) of the treated samples. Treatments: C (control, un‐coated beef slices), BA (coated beef slices by bacterial alginate), and AA (coated beef slices by algal alginate). Different letters within the same day (a, b) indicate a statistically significant difference (*p* ≤ 0.05).

pH changes of the stored meat are considered a main spoilage indicator (Ghanbar Soleiman Abadi et al. 2024). Figure 5a illustrates pH changes of the coated and uncoated beef thigh meat throughout storage period. There was no significant difference in the initial pH of all treatments on day 0, indicating same conditions of them. The pH values of the treatments changed as follows: C: 5.45 to 6.8, AA: 5.45 to 6.55 and BA: 5.45 to 6.16. The pH increase of the studied samples can be related to the endogenous and microbial proteinase enzymes that lyses sample proteins over the storage time, leading to the alkaline compounds production, such as ammonia, dimethylamine, and trimethylamine (Ghanbar Soleiman Abadi et al. 2024). Therefore, microbial growth inhibition can decrease these compounds, followed by the pH stability of the stored samples. In agreement to the microbial findings, the highest and lowest pH values belonged to C and BA groups during storage period, respectively. Elhadefa et al. (2024) reported that the pH value of the packaged minced beef meat with gelatin‐sodium alginate composite film increased from 5.4 to 5.66 on the 3rd, 7.46 on the 7th, 8.49 on the 10th and 8.8 on the 14th day of storage. The increase in the pH value indicates the degree of meat spoilage during protein breakdown. Another study showed the pH increase of the alginate coated beef meat from 5.59 to 5.64 on the 6th and to 6.51 on the 12th day of refrigerated storage (Smeti et al. 2025). In one study, the soy hull nanocellulose/sodium alginate/calcium chloride (CaCl_2_) hydrogel coating slowed down the pH changes of the coated beef slices compared with the uncoated samples during storage, indicating the protective effects of hydrogels on the stored beef. They concluded that microbial activity decomposed proteins to amine metabolites followed by increasing the pH value in agreement with the microbial analysis (Yu et al. 2024). Other studies found that by increasing storage time, the pH values of beef slices enhanced that can be related to the accumulation of volatile alkaline nitrogen substances (ammonia, primary, secondary and tertiary amines) produced by the breakdown of proteins by exogenous (bacteria) and endogenous proteinases (Kanatt 2020; Zhang et al. 2020). Previous studies reported that alginate sodium coatings resulted lower pH values of beef slices compared with the uncoated samples, which can be due to the decrease of microbial growth caused by coatings on beef surface (Zhang et al. 2021, 2022).

#### 3.5.2 TVBN

The total volatile basic nitrogen (TVBN) index is the main factor in the quality measurement of the meat samples. Bacterial catabolism of meat amino acids produces alkaline compounds, such as ammonium, and other nitrogenous volatile compounds that create off odor and flavor in the samples. The standard level of TVBN in consumable products is < 25 mg/100 g of the sample (Fernández et al. 2009). Figure 5b illustrates the TVBN values of the studied samples during the storage period. Initial analysis (day 0) revealed no significant differences (*p* > 0.05) in the TVB‐N values among coated and uncoated samples. During refrigerated storage, all samples exhibited progressive increases in the TVB‐N levels with a significant difference (*p* ≤ 0.05) among treatments. BA treatment demonstrated superior preservation efficacy, showing the lowest rate of TVB‐N accumulation. In contrast, the control group displayed the most pronounced increase in the volatile nitrogen compounds, and AA treatment showed intermediate TVB‐N values (Figure 5b). According to Figure 5b, uncoated (control group) beef slices showed the highest TVBN value (37.15 mg/100 g meat) compared with the others, and AA (25.85 mg/100 g meat) and BA (18.9 mg/100 g meat) treatments were in the next groups at the end of the storage period, respectively. According to the obtained findings, the TVBN value of the BA group never reached the allowable limit (< 25 mg/100 g of sample) at the end of the storage period. Yu et al. (2024) showed that the uncoated beef slices exceeded the standard limit of TVB‐N after 3 days at environment storage. Conversely, the shelf life of the coated slices with sodium alginate hydrogel was gradually extended at environment storage. This indicates that the sodium alginate hydrogel significantly (*p* ≤ 0.05) decreased the production rate of TVB‐N and elongated the shelf life of beef. In agreement with the obtained findings, another study reported that sodium alginate coated beef slices remained in the allowable limit of TVB‐N value after 12 days of storage period. This can be related to the preservative effects of alginate on the beef surface (Zhang et al. 2022).

### 3.6 Sensory Analysis

**FIGURE 6:**
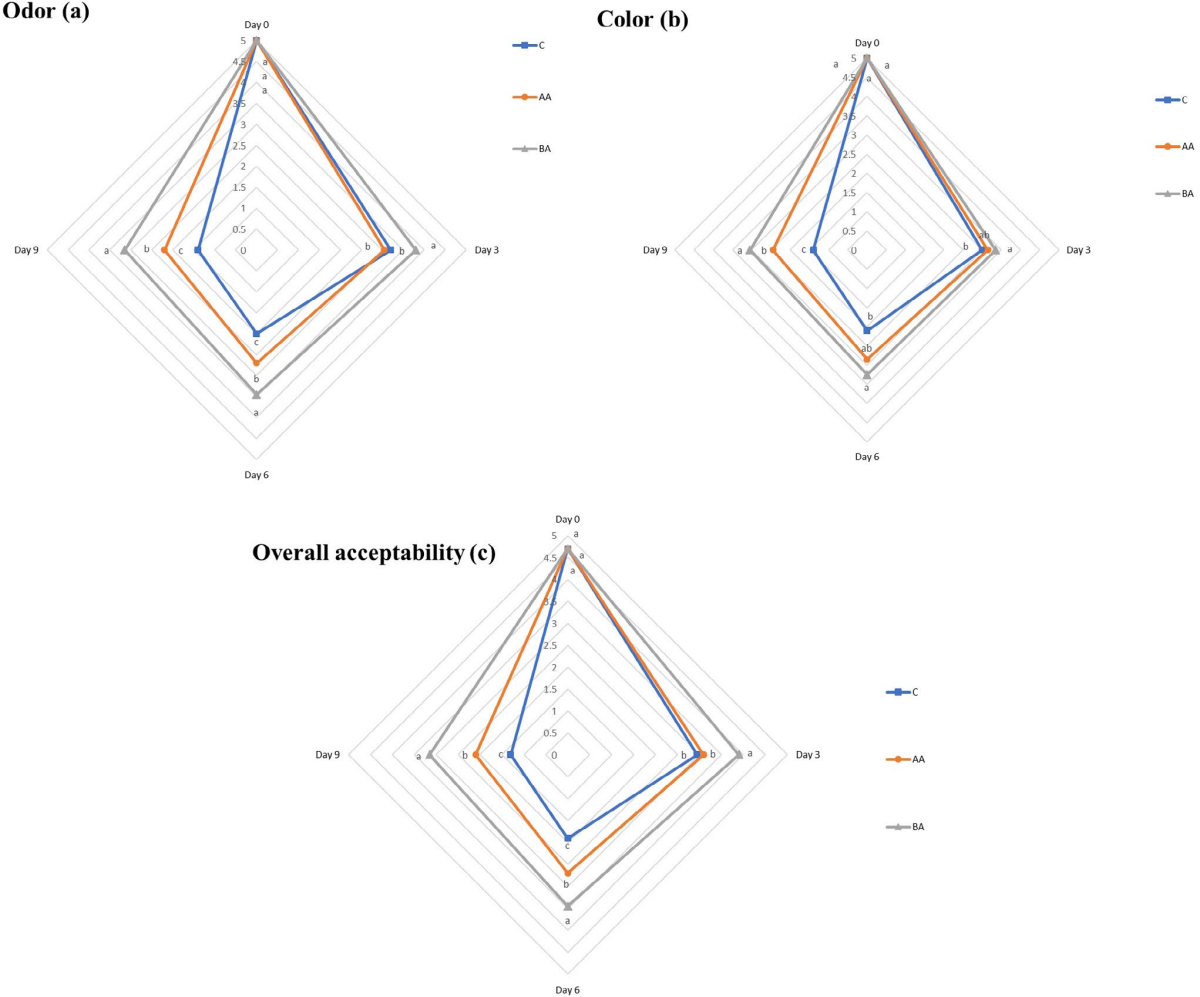
(a–c) Changes of odor (a), color (b), and overall acceptability (c) features of the treatments. Treatments: C (control, un‐coated beef slices), BA (coated beef slices by bacterial alginate), and AA (coated beef slices by algal alginate). Different letters within the same day (a, b, c, etc.) indicate a statistically significant difference (*p* ≤ 0.05).

Sensory features (odor, color, and overall acceptability) of the studied samples were illustrated in Figure 6a–c. The initial score of the sensory characteristics of all treatments was 5 on day 0. By increasing the storage period, sensory features significantly (*p* ≤ 0.05) decreased in all treatments. Consistent with the bacterial and chemical analyses, BA treatment significantly (*p* ≤ 0.05) demonstrated superior performance across all sensory parameters over storage time. According to Figure 6a–c, the highest sensory scores belonged to the BA group, followed by AA and control, respectively. In other words, the higher preservative effects of the BA coatings led to the higher sensory scores of the coated meat over the storage time. Control samples earned the lowest scores and became unusable (< 3 score) on the 3rd day of the storage period. But BA treatment remained consumable (> 3 score) in odor (Figure 6a), color (Figure 6b), and overall acceptability (Figure 6c) features until the end of the storage period. Vital et al. (2016) reported that the alginate‐based edible coatings significantly (*p* ≤ 0.05) delayed the weight loss and lipid oxidation in the coated beef for up to 7 days of storage period. Texture and sensory analyses revealed that coated meat was redder and more tender compared with the uncoated ones. Elhadefa et al. (2024) observed that gelatin‐sodium alginate composite film preserved sensory properties (odor, color, appearance, and overall acceptability) of the wrapped minced beef meat during 14 days of storage period. So, color scores were acceptable until the 7th day. Also, the rancid odor of the wrapped minced beef meat was revealed after the 14th day of storage time.

Another study showed that the color and odor changes of alginate sodium‐coated beef slices became unacceptable after 15 and 12 days of storage. According to the overall acceptability scores, the alginate sodium coating could enhance the shelf‐life of coated beef slices until 11 days of storage under refrigerated conditions (Zhang et al. 2021). They reported that these findings were in agreement with the microbial and chemical analyses of the coated samples.

## 4 Conclusion

Bacterial alginate was harvested from *Pseudomonas aeruginosa* in laboratory conditions. FT‐IR analysis confirmed the successful synthesis of alginate. Coating and film formation properties of bacterial alginate (BA) with algal alginate (AA) were compared. It is concluded that BA and AA showed no similar shapes and properties and exhibited significant differences (*p* ≤ 0.05) in the evaluated characteristics. According to the SEM images, bacterial alginate particles exhibited a rough surface, irregular polygonal structures with sharp sides, and corners in comparison with AA. The mechanical features (TS, EB, YM) of AA films were significantly (*p* ≤ 0.05) more superior to BA films. It was revealed that BA coatings were more efficient than AA coatings in the shelf‐life improvement of the coated beef thigh slices over the storage period. It can be related to the more antimicrobial and adhesion properties of BA than AA. Finally, it is concluded that BA treatments are the strongest groups in the shelf‐life enhancement of the refrigerated beef slices among other treatments in coating forms. Therefore, considering the mechanical characteristics of AA and the barrier and preserving properties of BA, it is recommended to use a mixture of these two alginates in an appropriate ratio in food packaging and formulation for future research.

## Author Contributions

**Behnaz Bazargani‐Gilani:** conceptualization, supervision, methodology, formal analysis, writing – review and editing, funding acquisition. **Ali Goudarztalejerdi:** investigation, methodology, conceptualization, formal analysis, software, validation, visualization. **Leila Soleiman Garmabaki:** investigation, validation, data curation, software, resources, methodology.

## Funding

The authors have nothing to report.

## Conflicts of Interest

The authors declare no conflicts of interest.

## Data Availability

Data will be made available on request.
